# Complete Genome Sequence of *Shewanella* sp. Strain TH2012, Isolated from Shrimp in a Cultivation Pond Exhibiting Early Mortality Syndrome

**DOI:** 10.1128/MRA.01703-18

**Published:** 2019-03-28

**Authors:** Piyanuch Wechprasit, Muthita Panphloi, Siripong Thitamadee, Kallaya Sritunyalucksana, Anuphap Prachumwat

**Affiliations:** a Center of Excellence for Shrimp Molecular Biology and Biotechnology (Centex Shrimp), Faculty of Science, Mahidol University, Bangkok, Thailand; b Department of Biotechnology, Faculty of Science, Mahidol University, Bangkok, Thailand; c Shrimp-Pathogen Interaction (SPI) Laboratory, Animal Biotechnology Research Unit, National Center for Genetic Engineering and Biotechnology (BIOTEC), National Science and Technology Development Agency (NSTDA), Pathum Thani, Thailand; University of Rochester School of Medicine and Dentistry

## Abstract

Here, we present the complete genome sequence of a Shewanella isolate, TH2012, from a shrimp pond in which shrimp exhibited early mortality syndrome (EMS)/acute hepatopancreatic necrosis disease (AHPND). The complete genome of TH2012 has a prophage-like element and a number of potential virulence factors, making TH2012 a possible contributing factor to EMS outbreaks.

## ANNOUNCEMENT

Early mortality syndrome (EMS) and its component acute hepatopancreatic necrosis disease (AHPND) have caused massive production losses in farmed shrimp since 2009 (1, 2). Unique Vibrio parahaemolyticus isolates that produced Photorhabdus insect-related-like toxins in V. parahaemolyticus (Pir^vp^ toxins) were found to be the cause of AHPND (termed VP_AHPND_ isolates) (3–5), and such toxins have recently been found in other Vibrio species (6–8). Along with other Thai VP_AHPND_ isolates, the bacterium Shewanella sp. strain TH2012 was obtained from hepatopancreatic tissue of diseased shrimp in an EMS/AHPND outbreak pond in 2012 (9). TH2012 was tentatively identified as a member of the genus Shewanella on the basis of both high sequence similarity of its partial 16S rRNA gene to matching sequences from the genus and highly matching biochemical profiles of API 20E stripe tests (bioMérieux). Found predominantly in aquatic environments, several Shewanella species are opportunistic pathogens of aquatic species and humans (e.g., references 10 through 12). It is not currently known whether TH2012 is pathogenic to shrimp. The isolate was revived directly from cryopreserved glycerol stocks for aerobic overnight culturing at 30°C in tryptic soy broth (TSB) supplemented by 1.5% (wt/vol) NaCl, and the overnight culture was used as the inoculum for the subsequent culture before total genomic DNA extraction using the standard phenol-chloroform method (13). DNA concentration and purity were measured by the PicoGreen method (Invitrogen, USA) and by gel electrophoresis before construction of a 20-kb library for sequencing with PacBio RS II technology on one single-molecule real-time (SMRT) cell (Macrogen, South Korea). The high-quality filtered subreads (with subread lengths of ≥500 bp, polymerase read lengths of ≥100, and polymerase read qualities of ≥0.8) were assembled *de novo* by Hierarchical Genome Assembly Process 3 (HGAP3) (14) using default parameter settings (with a seed read length of ≥6,000 bp). Complementary ends of assembled contigs were joined and trimmed to form circularized contigs that were subsequently polished with Quiver using a default parameter setting (14). Basic Local Alignment with Successive Refinement (BLASR) (version 1.3.1.124201; –bestn of 1 and minSubreadLength of ≥1,000 bp) (15) was used to map subreads against the circular polished contigs with two different positions of the first base pair location for each contig. To verify contig circularity, relatively even read coverages within the joined regions and along the contigs were observed with the Integrative Genomics Viewer (IGV; version 2.4.18) (16). Prokka (version 1.11) (17) was used to predict open-reading frames (ORFs), tRNAs, and rRNAs, relying on Prodigal (version 2.6) (18), ARAGORN (version 1.2) (19), and barrnap (version 0.7; https://github.com/tseemann/barrnap). Pfam domains were predicted by an hmmscan search against Pfam-A.hmm with an E value of <0.01 (20), whereas clusters of orthologous groups (COGs) were obtained by a BLASTP (21) search against the UniProt database of eggNOG (version 3.0) (22), with top hits for E values of <10^−3^. Transmembrane helices were predicted by TMHMM (version 2.0c) (23) with default parameter settings. ORFs were additionally annotated by BLASTP and BLASTX searches (21) against the NCBI nonredundant (nr) database (21). PhiSpy (version 2.3) (24) was used to detect prophages with a default parameter setting, using either the generic test set or Shewanella oneidensis and Escherichia coli as closely related organisms. Similarly, genome annotation was performed using the Integrated Microbial Genomes Expert Review (IMG/ER) platform (DOE Joint Genome Institute) (25).

The sequencing resulted in 157,485 high-quality filtered subreads with an average length and an *N*_50_ value of 7,967 and 12,011 bp, respectively. The assembled genome contained 4,858,998 bp (258× coverage; 54.81% G+C content) with a single circular chromosome of 4,808,629 bp (204× coverage; 54.88% G+C content) plus a circular plasmid, pSTH1, of 50,369 bp (750× coverage; 48.1% G+C content) (Table 1). The whole genome contained 4,303 predicted genes, 4,176 ORFs, and 127 RNA genes, with 4,109 ORFs on the chromosome (88.04% coding density) and 67 on plasmid pSTH1 (89.67%). The majority of the ORFs were assigned putative functions, leaving 1,038 genes (24.86%) coding for hypothetical proteins. No *Pir^vp^* sequences of VP_AHPND_ (3, 26, 27) or *Pir^vp^*-like sequences of Shewanella violacea (GenBank accession numbers WP_013050436 and WP_013050437) were found by exhaustive searches, and there was no significant nucleotide similarity of pSTH1 to the *Pir^vp^* toxin-carrying plasmids, pVA1 (3), pVA1-3 (26), and pVH_vo_ (27). On the other hand, the genome contained one putative prophage-like element of 3.76 kb, in addition to several putative hemolysins, chitinases, and proteases that have been implicated in the virulence of some Shewanella isolates (12, 28). Based on the genomic information, further investigation is needed on the TH2012 isolate’s pathogenicity to shrimp, on the possibility that it can act synergistically to potentiate the virulence of VP_AHPND_, and on development of specific detection methods to determine its prevalence in shrimp ponds.

**TABLE 1:** Genome statistics of *Shewanella* sp. strain TH2012

Attribute	Value	% of total
Genome size (bp)	4,858,998	100
DNA coding genes (bp)	4,322,791	88.96
DNA G+C content (bp)	2,663,040	54.81
No. of DNA scaffolds	2	
Total no. of genes	4,303	100
No. of protein-coding genes	4,176	97.05
No. of RNA genes	127	2.95
No. of genes in internal clusters	775	18.01
No. of genes with function prediction	3,138	75.14
No. of genes assigned to COGs^*a*^	2,902	67.46
No. of genes with Pfam domains	3,722	89.13
No. of genes with signal peptides	616	14.32
No. of genes with transmembrane helices	1,088	26.05

a COGs, clusters of orthologous groups.

### Data availability.

This whole-genome shotgun project has been deposited at GenBank/EMBL/DDBJ under the accession number CP020373 and BioProject number PRJNA378141 and at IMG/ER under Genomes OnLine Database (GOLD) identifier Gp0206473. The version described in this paper is the first version, CP020373.1. The associated PacBio RS II subreads are available under the SRA accession number SRR8549885.
